# Book Reviews

**Published:** 1832-07

**Authors:** 


### CRITICAL ANALYSES.

Quæ laudanda forent, et quæ culpanda, vicissim,

Illa prius, cretâ; mox hæc, carbone, *notamus.*—Persius.

*Les Lois de la Revulsion, etudiées sous le Rapport Physiologique et Therapeutique.* Par J. C. Sabatier (d'Orleans), Docteur en Médicine de la Faculté de Paris. *Mémoire couronné par la Société Medico-pratique de Paris,* 17 *Octobre,* 1831.

This pamphlet originated in the following question proposed by that Society: "Quelles sont les lois de la revulsion, les secours que la therapeutique peut en attendre; et par consequent les avantages et les inconveniens des revulsives suivant les cas auxquels on les applique; l'opportunité de leur emploi?" The intimate relations between the most distant parts of our frame, where even dissection often fails to trace any nervous communication, will at once shew the importance of the subject.

Revulsion, the derivation of a morbid accumulation of fluid from one part to another, is a principle much employed by the advocates of the humoral pathology. They generally carry it so far as to make the evacuation at a remote part from the seat of disease: to relieve the head, they would bleed in the foot; in pleurisy, they would open a vein in the opposite arm to the side affected. Of late these refinements have fallen into disuse: however, much of the practice of medicine, both in acute and chronic diseases, hinges on this principle. Thus, in the congestive inflammation of internal organs, blisters, or stimulating liniments, are applied to parts less important to life, and the more important ones are thus relieved. The notions of the ancients on the doctrine of humours, proceeded in a great degree from their limited knowledge of physiology and the circulation: thanks to the discovery of our illustrious countryman Harvey, those false ideas, which time had in a measure sanctioned, are no longer maintained.

The laws of revulsion may be said to include all the sympathetic relations of our organization, without extending to the sympathetic affections of mind, whose operation is generally extended to external objects; the sympathy of body is referable only to itself, and therefore involved in obscurity. Revulsion, though an old doctrine, is still involved in darkness. The Greeks had four kinds, to correspond to the humours. Hippocrates established, as a principle of revulsion, venesection in the vicinity of disease.

Without entering into those views, which are nearly obsolete, we shall proceed to consider the book before us. The author, who candidly confesses himself alike ignorant of the elements of the phenomena and laws which regulate revulsion, thus defines it:

"A vital phenomenon, by virtue of which a morbid disposition affecting one organ is gradually diminished, or promptly dissipated; under the influence of a certain modification which an operation of art and sometimes also of nature excites and maintains on one or more organs, for a certain time, more or less remote, and having with the first relations more or less direct, naturally or accidentally established."

This, however, leaves the subject of revulsion, which we can only know by its effects, still involved in darkness; but the author, it appears, is proceeding from effects to causes. All phenomena occur in the living organ, he says, by virtue of certain conditions; and those necessary for revulsion are the previous existence of diseases. The following principles are laid down.

"1st. Irritation is the primitive and principal form of the greatest number of diseases.

"2d. There is but a very small number in which irritation exists only in a secondary form.

"3d. There are some in which irritation does not exist at all, into the composition of which it never enters either in a primitive or secondary form."

Thus disease, which is but the deranged function of the living organization, depends on the excess or diminution of that property indispensable to living matter, sensibility or excitability. The limitation which our author prescribes to sensibility, which he considers as a vital property, different from that tactile sensibility, which indicates the healthy condition of the cerebro-spinal system, does not appear to us well defined. When we consider that life is but a succession of movements excited by reason of the impression which the several organs receive, and that physical sensibility is the last term at which we arrive in the study of the phenomena of life, we are inclined to think that the author is confounding sensibility with irritability. Sensibility is that vital property by which every part of our organization is capable of feeling or being excited, and when the nervous influence of relation exists, excites sensation; but when this influence is suspended, particularly in man, (for in the classes Polypi and Infusoriæ sensibility exists, though no trace of a brain or nervous system can be discovered,) we would be inclined to suppose that sensibility no longer existed. But the author says, that, though the nervous influence be destroyed, sensibility remains, and that sensation is only altered in degree. This sensibility he supposes to be independent of the nervous system, and to exist by "the assemblage of those conditions which give vitality to a part." He leaves us, however, in ignorance of the manner by which this is effected. When sensations are abolished, he states that the apparent phenomena of excitation discover themselves. The cases which he adduces in support of this view are not quite conclusive. One is, that a paralysed limb deprived of sensation and motion, is capable of being affected by a blister in the same manner as a sound one. Here it is not more unreasonable to suppose the existence of sensation and motion, though not in a degree capable of producing the ordinary manifestations, than to imagine the existence of a property for which we can offer no strict physiological reason.

It appears, indeed, a contradiction in terms, to suppose that where all tactile sensibility is lost, excitation is capable of calling it into existence. Sensibility, like all other vital properties, is referable only to living tissues, and the nervous is the only one to which we can refer it. The nervous system, divided as it is into the cerebro-spinal and ganglionic, the former presiding over the functions of relation, the latter over organic life, incline M. Sabatier to suppose that, where the functions of the nerves of relation are suspended, as in a paralysed limb, the nerves of organic life are capable of being stimulated by excitation, which he considers the *sine qua non* of sensibility.

The various effects of excitation, producing first irritation and its phenomena, until at length it terminates in inflammation, next engage our author's attention.

After noticing the effects of revulsive agents, which may be either physiological excitation, complete irritation, or inflammation, he divides his subject into the three following parts.

"1st. The most general means by which revulsion is effected, and their most simple mode of action.

"2d. The conditions necessary that this action may take place.

"3d. The conditions by virtue of which the phenomena of revulsion are completed."

The accuracy and perspicuity with which he proceeds to elucidate the means by which revulsion may be effected, can only originate with a mind well acquainted with the subject; a subject which has, and still labours under the empirical notions of the past ages. His distinctions into external and internal excitants, either as applied by art or nature, producing either simple excitation, irritation, or inflammation in the ratio of their application, goes far to establish, on the clearest physiological principles, the doctrine of revulsion. The action of artificial revulsives is generally ascertained by their impression on the parts with which they are in contact.

"1st. The external means are sinapisms and blisters, to produce superficial irritation.

"2d. Moxa and the actual cautery, to excite deeper irritation, accompanied with inflammatory phenomena.

"3d. Leeches and cupping glasses, to excite irritation or simple local excitation, with congestion of capillaries.

"4th. Electricity and galvanism, to produce direct excitation of the sensibility of a part, capable of being communicated to others.

"1st. The internal means are laxatives and mild purgatives, causing more or less excitation of the digestive mucous membrane.

"2d. Drastic purgatives, too powerful or too often repeated, causing irritation, with inflammation of one or more portions of that membrane.

"3d. Sounds or bougies, covered with certain substances, introduced into the urethra, or liquids injected into that canal, to promote irritation or a special inflammation upon other mucous membranes than that of the digestive canal, determining a suppressed morbid secretion.

"4th. Mercury used either internally or by friction; certain diffusible stimuli or medicines, which by absorption produce excitation or irritation."

The internal means which nature employs are certain acute diseases developed during the course of other diseases. The author justly observes, that revulsion effected by art is slower in its operation than that which nature excites, though safer in its results: indeed, it rarely occurs that we can ever consider a revulsion by nature completely exempt from danger. Strange as this may appear, it is an observation sanctioned by experience.

Revulsion is sometimes confounded with repulsion; in the former, the morbid phenomena succeed, in the latter they precede the new disease. Inattention to this point has often ascribed to repulsion the production of disease, which should be ascribed to other causes; as when pneumonia occurs during a cutaneous affection, a species of revulsion is effected; the vesicles subside during the progress of pneumonia, and again reappear as the pulmonic attack is subdued. The revulsive effects of acute diseases have been considered in a twofold light by our author, where the quick development of a violent irritation of one organ removes that of another, in which the progress and development of irritation were more tardy, or where the new disease produces such an augmentation of the general sensibility as to accelerate the march of the original affection.

The divisions which Sabatier lays down, according as revulsive agents operate, are of the greatest importance, and founded on the closest practical observation.

1st. From skin to skin.

2d. From skin to mucous membranes.

3d. From skin to other organs.

4th. From mucous membranes to mucous membranes.

5th. From mucous membranes to other organs.

6th. From organs to organs.

To produce the revulsive effects on these different surfaces, a degree of sensibility is indispensable, and in proportion to the healthy condition of the cerebro-spinal and ganglionic systems will be this effect.

We next come to the consideration of those conditions where the local action of revulsives excite immediate sensible effects; and here we must suppose the anterior existence of sensibility, which, though sometimes concealed from our researches, is rendered manifest by excitants, where the sensibility of parts enjoying vital properties is not suspended by some unknown cause; for we find parts possessing different degrees of vitality vary so much, that we can account for it only by supposing a temporary suspension of their vitality, (a thing almost impossible,) or the translation of its sensibility to more remote parts. In this case the author supposes the parts are endowed with new properties, by virtue of which they resist the influence of the most energetic agents. In support of this view, he adduces a few cases.

A patient was attacked with a violent neuralgic affection of the spermatic cord, accompanied with other nervous symptoms; amongst the remedies employed, was morphine, by the *methode endermique:* and in order to expose a large surface, a heated iron was applied to the skin of the perineum, without producing any effect, the eschar which generally follows such an application did not appear here, and the skin retained its usual appearance.

Another patient was attacked with erysipelas of the face; the symptoms became very severe; after large bleeding, recourse was had to blisters and sinapisms, but without any effect, the epidermis, on the removal of the blister, was not even detached; it was only a little moist. Though, from this, it would appear that there are certain states or conditions of our organization in which the most powerful irritants cannot produce any sensible effect, yet the author very justly observes, that, to determine this *a priori* is impossible: experience may foresee it, observation only can establish it.

These observations do not apply to internal revulsives; for we often find that revulsion takes place without manifesting any internal irritation. When, from some cause, the sensibility is much diminished, the most powerful irritants seldom produce any effect.

" A woman labouring under arterial hemorrhage was brought into hospital; on the stopping of the hemorrhage, the pulse was scarcely perceptible; blisters were applied to the hams, and allowed to remain on for three hours; they were then removed. The skin did not exhibit the slightest tinge of red, nor was there any pain felt."

In diseases characterized by excessive weakness of the general sensibility, the most active irritants seldom produce any sensible effect. From what has now been urged, our author thinks he is justified in establishing the following conclusions.

" 1st. Without sensibility, we cannot suppose any means capable of operating revulsion.

" 2d. In order that the agents determine on the parts where they are applied immediate effects, it is necessary that sensibility be within certain limits, on either side of which their effects are not produced.

" 3d. The local action of revulsive agents will be in proportion to the healthy state of the parts."

In treating of the various modifications which the local application of revulsive agents produce, the author divides them into the following order: physiological excitations, irritations, and phlegmasiæ. The conditions necessary to the production of these depend on sensibility, and are maintained by the sympathetic action of remote parts. Sympathy, that first feeling of social life, and upon which the laws of revulsion hang, may be either universal or partial. Hunter divides partial sympathy into remote, contiguous, and continuous; as the pain in the shoulder, when the liver is affected, is an instance of remote sympathy; affections of the lungs with the chest, the contiguous; and the uninterrupted sympathy of parts, as the continuous. The manner in which the author treats this subject, if not quite explanatory of the *modus operandi*, at least bears the impress of a mind well acquainted with its phenomena.

In every form of disease, as well as in health, there exist such close and intimate relations between all the parts of our frame, that one part cannot be affected in structure or function without involving all or most of the other organic functions of life. This connexion of different organs is termed the sympathetic phenomena, and the power which presides over them is called sympathy, one of the distinguishing characteristics between brute and organic matter. That the movements of an animal and a piece of mechanism may be influeneed by parts immediately in contact, is a circumstance well known in the physical as well as mechanical world; but that parts the most remote should maintain a connexion, and exercise functions indispensable to life, without any visible agent, is what must excite our admiration.

The divisions of sympathy which the author establishes are full of interest, and cannot fail to engage the attention of all who have embraced the profession from other than worldly motives. But, as regards the principle of sympathy, we cannot help thinking that his views rest rather upon hypothesis than fact. "Sympathy (he says) is independent of all sympathetic action, and may exist without manifesting itself." This may be so, but it can only be known by its effects. Every sympathetic action supposes two points; one the source whence it emanates, the other the limit of its operation. Many theories have been urged to explain the operation of sympathy as transmissible either by membranes, cellular tissue, or vessels; but late investigations lead to the belief of the nerves being the medium of communication. "Sensibility, the author states, is necessary to sympathy; it can only exist in a sensible tissue, which is the nervous; ergo, he says the nervous tissue is the agent of sympathy." Our connexion with the external world is maintained by two orders of nerves, the cerebro-spinal and ganglionic; the former communicating impressions directly to the brain, the latter communicating impressions to the first, and by their intermedium to the brain. But there are states of our organization where the impressions conveyed from the second order of nerves are so weak as not to excite any sensation: this does not necessarily imply the non-existence of sympathy; for in the healthy condition of life, the ordinary impressions sometimes pass unnoticed. "Sympathy," which he defines to be "a disposition naturally or accidentally established between two or more organs, by virtue of which one or each become susceptible of being influenced by the more or less evident modification which the other experiences," can only be said really to exist where the relations of one or more organs are so inseparably connected as to be evident to our senses. This intimate connexion may, and does exist, though the bond of union be still unknown; and, whether we ascribe it to the Archæus of Vanhelmont, the Anima of Stahl, the invisible nerves of others, or the imponderable fluid of later writers, as a fact, its existence is admitted by all, though inexplicable by any. "Nos latet æternumque latebit minima illa ac subtilis; non solum a sensibus sed ab humanæ mentis acie prorsus remota."*

The classification of the sympathetic phenomena of health and disease into physiological, pathological, and therapeutical, though advantageous in the arrangements of facts, is not free from error. Thus he states, that certain pathological sympathies may be nothing but physiological ones exaggerated; certain therapeutical sympathies are developed only by means of morbid sympathies. "Frequently the morbid state has modified one of the organs in such a manner, that revulsive agents become necessary, in order that the relations of physiological sympathy which existed between them be re-established. In this case, the exercise of sympathy is at the same time both therapeutic and physiological." Thus does nature or disease easily confound the most subtle divisions of the physiologist or pathologists.

The author now proceeds to consider the effect of sympathetic irritation, which is always accompanied with local phenomena, but which do not, in every case, exhibit the sympathetic: hence the necessity of applying revulsive agents to parts possessing the strongest sympathetic relations. The impression which the application of means to one organ makes upon another is what he denominates sympathetic irradiation, and he thus exemplifies it:

"The impression is transmitted to the principal nervous centre by the intermedium of the organ, the limit of the sympathetic irradiation; or rather the nervous centre is itself the limit of this irradiation, and re-acts upon that organ by a marked preference."

The importance of this part of the subject, and the enlightened and accurate views of the author, not only justify, but render necessary, our quoting largely from the original, in order to comprehend fully the accuracy of his deductions.

"The point from which the sympathetic irradiation proceeds is the organ upon which the revulsive agents have exercised their action; the limit of this irradiation may be an organ identical, analogous, or different. The most numerous sympathies are observed between identical organs, and next between those which are analogous." Organs placed symmetrically or parallel in the lateral portions of the body, have peculiar sympathetic relations. The works of the ancients are replete with examples of this kind. Morgagni relates the case of a child, subject for years to convulsions; the extremity of one hand being particularly affected, the slightest efforts to extend the fingers of which brought on, in the sound hand and fingers, convulsive twitchings. Many such cases might be adduced in proof of the sympathetic relation between parts

* Opera Baglivi Lugd., cap. 2, sec. 5.

in the corresponding portion of the body. An extraordinary case of this kind is mentioned by BARTHEZ: a patient had the right arm paralysed; a blister applied to it produced its effect on the corresponding part of the left arm, which became red, and was affected with acute pains during the application of the blister. Nevertheless, the paralysis of the right arm was removed, and attacked the left. A blister applied to this arm had an effect similar to the one applied to the right: yet the paralysis abandoned both arms, and the blisters had nothing more particular in their effect. Our author explains this, by stating that the pathological state was the cause of that remarkable sympathy which the therapeutical agent had excited." To establish this sympathetic irradiation, a certain aptitude is necessary, or, in other words, a state or condition of the organic functions of the economy, which originate the natural sympathetic phenomena. The author extends this to the sympathies developed by revulsive agents, and observes, "It is from certain modifications impressed on an organ by the morbid state which they induce; they may be independent of physiological sympathies."

If we compare the sympathies arising from irradiation with those physiological ones which every body possesses, we shall find them differ much; the former being in the ratio of the excitation, whilst the latter, in general, correspond to that of the organ with which they sympathize, as the sympathy of the iris is determined by that of the retina, &c.; on the contrary, the sympathy of revulsion is determined by an inverse modification to that of the organ primarily affected: thus he states, that "secondary artificial excitation, far from aggravating the morbid phenomena which have determined the primitive excitation upon another part, on the contrary relieves it, and removes those with the cause which has produced them."

The sympathy of excitation can only be produced by establishing in the sympathizing organ a sensibility different from that of the organ primarily affected, which checks the phenomena originally produced, or by relieving the organ primarily attacked of its excitation, by causing a derivation to itself.

The caprice with which revulsive agents act in the different states both of health and disease, is still involved in mystery. It has been supposed that the active principle was for a time suspended: the author's views on this point are so important that we make no hesitation in quoting the following passage from the original.

"There are cases, where the local effect of revulsive means is at first insensible, by reason of the excessive exaltation of sensibility upon an organ, which very soon extends itself sympathetically to other points, and may even become general. In this case, the organs sympathetically affected, sometimes even the entire organism, are found to suffer violently from spasm, which renders them for the time incapable of answering immediately, each in its own manner, to the excitations which are communicated to them. Then, even the most powerful agents, for this reason, cannot exercise their immediate local effects upon certain parts of the economy where they are applied, though they make an impression, as we shall shew. What occurs then? It is that here the exercise of therapeutic sympathy precedes the appearance of the local phenomena. It is that sometimes by its sole influence, sometimes by the concurrence of other means, the organ whose sensibility, vividly increased, extended to others, is by degrees brought back to the standard of healthy sensibility; then the morbid sympathies which had been excited cease, then the other organs return by degrees to their natural state; then also the local phenomena begin to manifest themselves, whose extent and energy are in the direct ratio of the impression received. We see that, in order to produce them, it was necessary that the therapeutic sympathy subdued at first the morbid sympathy."

In dwelling thus on the subject of sympathy, we hope that the importance of the subject and the originality of the author's views are a sufficient apology. In the short space we have allotted to it, it would be impossible to do justice to its merits: we refer the reader to the original.

Having followed revulsion through all its changes, and pointed out the different actions which an organ by its influence undergoes, he now proceeds to consider the modifications which the sympathetic action excites in the affected organ, determining its return to health. The sympathetic phenomena of revulsion are always regulated by the condition of the organ, and the epoch of the disease. An organ may become hypertrophied, or its function changed and a morbid production be the result; or there may be effusion of pus or serum into a cavity or the cellular tissue: in these cases revulsion acts by suspending the morbid operation impressed by it; the author says, a new activity is impressed on the vessels which penetrate the organ. Then, under the influence of a new mode of sensibility, an absorbent action is established, either in the centre of the hypertrophied organ, and to a certain extent at its expense; or of the substances developed in its tissue, and which losing their activity in developement, become in some sort foreign bodies, and by this subject to absorption, which by little and little removes them."

Thus it appears, as the author asserts, that the last acts in the chain of revulsion are resolution and absorption, by which an organ may be restored to its healthy condition. Experience proves that the morbid secretion of an organ is diminished in proportion to the analogy that exists between the organ we endeavour to stimulate and the one primarily affected. This is a point, though resting in a great degree on the humoral pathology, full of practical importance; and in whatever light we consider the diseased product, whether as a morbid secretion or an imbibition of fluids into the cellular tissue, the possibility of checking its vital properties, and thereby reducing it to the state of a foreign body, which the common principle of nature will endeavour to rid herself of, is in itself a subject that merits particular attention. A theory of sympathetic metastasis has been urged by other writers, which differs from this of our author. Barthez, its ablest advocate, admits the possibility of this metastasis occurring through the cellular tissue; he alleges that it is this that obtains between the cellular tissue which penetrates the viscera and that of the extremities, and quotes from Lieberkühn, in support of it, a case of pulmonary œdema, where "foot baths determined to the lower extremities water infiltrated into the cells of the lungs." A little reflection will be sufficient to shew those who are at all acquainted with the structure of the human frame, that this direct metastasis from the lungs to the extremities by the cellular tissue is, if not impossible, at least very improbable. Our author's explanation seems more consonant to the principles of physiology or therapeutics: he supposes the application of caloric under the form of the footbath to excite in the first place an absorption of the effused fluid, and in the second a deposition from the circulating mass, on the lower extremities. This, though it may be an approximation to truth, still leaves the subject in great obscurity.

Bleeding, both local and general, which has been long considered as the most powerful revulsive, and which the obscurity that overhung the laws of revulsion could alone justify, is viewed by the author in a new and interesting light. Its object being the relief of a particular organ, or the system at large, acts in general, by reducing the circulating mass of blood, the natural excitant of sensibility, and thus establishes an equilibrium of the deranged functions, which by a loose habit of thinking has been considered as revulsion. That it does not act as a revulsive, appears from the following: a cerebral congestion succeeds a suppression of the menses; a bleeding in the foot relieves the congestion, the menses reappear. Similar results would have followed a bleeding at the arm: the following phenomena occur.

1st. A rapid and abundant loss of blood.

2d. A diminution of the general sensibility, and consequently of the cerebral excitation.

3d. A diminution and cessation of the afflux to the brain; and then,

4th. A return to the healthy state, both of that organ and uterus.

5th. The re-establishment of the equilibrium of the functions of both organs.

6th. A return of the menses.

Thus general bleeding, which reduces the afflux of fluids to an organ, and thereby its sensibility, cannot be considered as a revulsive agent. In like manner the disappearance of a congestion or an œdema after bleeding, is the result of its mechanical effect, rather than its revulsive power. With respect to the effect of leeching, he thinks that it is in the depletion which they occasion, and not the local congestion, that their efficacy lies.

From all that has now been advanced, revulsion it appears is not in itself an act; it is but the result of the therapeutical means we employ to obtain it. "Revulsion is the result of a complex therapeutic operation, to the accomplishment of which many phenomena concur."

Thus we close our notice of the laws of revulsion; and if the author has not cleared away the lumber under which it has for ages lain, he is entitled to our greatest respect for the talent and industry which he has bestowed on a subject of such vital import. In our next we shall notice the effects which therapeutics derive from it, and the evils or advantages of revulsion.

---

*Observations in Surgery and Pathology, illustrated by Cases, and by the Treatment of some of the most important Surgical Affections.* By William James Clement, Surgeon.—8vo. pp. 230. Whittaker and Co., and Highley, London; and Watton, Shrewsbury.

It is a relief in this age of philosophy and in this land of philosophers, occasionally to get clear of baseless speculations, and to find ourselves travelling along the humble path of practical observation; for, although the pen and the tongue may move more freely when unshackled by the necessity of proving their assertions, than when confined by the fetters of facts, still the judgment becomes bewildered in the regions of imagination, and can neither abandon itself entirely to the blandishments of fiction nor enjoy the tempered society of sober truth. Accustomed as we are to ponder upon theories falsely so called, and to search in vain for the principles of hypotheses which are any thing but Ὑπό θεματα, it is no slight gratification occasionally to find some facts given, unsophisticated by speculation, on which we are at liberty to theorize for ourselves, and materials afforded whence we may assume principles for our own hypotheses. Such, or nearly such, are the "Observations in Surgery and Pathology" published by Mr. Clement, which are evidently the fruits of an extensive practice carried on by an able surgeon. Much good might result to the science of medicine did surgeons acustom themselves to register cases with the care that our author seems to have done; for from such materials only can general doctrines and precepts be legitimately deduced, and persons frequently permit themselves to generalize on far too slender an accumulation of facts. It is not that we meet in this volume with any brilliant discovery, or any very important improvements in practice, that we may approve of its contents; but because it details with simplicity some of the most important and some of the most common (and the still more important because the more common,) disorders to which the human frame is subject, and records unaffectedly the success of treatment, which should be always borne in mind, and should be always familiar to the well-educated surgeon, but which students are too apt to pay much less attention to than to some of the more striking but less important, because less frequent, operations of surgery.

Such is, in our opinion, the chief merit of this publication; and, if any fault need be found with it, it will be in the extreme to which common observations are carried, which sometimes sound rather common-place; such for example, is the following:

"I attribute the ill success which too commonly attends operations for strangulated hernia, to the great delay before performing them; thereby bringing a good and perfect operation into disrepute, whereas there is nothing faulty in the operation itself, when properly and carefully performed." (P. 55.)

And again, the following is a truism which no one would deny.

"I have seen one case of stone occurring in an adult female, which, after great difficulty, was removed through the urethra; but, either in consequence of the extreme dilatation of the canal necessary to admit the passage of the calculus, or from some injury done to the neck of the bladder in extracting it, the woman could never afterwards exercise any voluntary power of retaining her water, but was constantly subjected to a stillicidium urinæ. This case, therefore, proves that, when the female urethra is mechanically dilated beyond a certain extent, the power of retaining the urine is as much endangered as by a division of the canal and the sphincter of the bladder with a cutting instrument." (P. 191.)

We like the first essay, viz. "*the critical inquiry into the different opinions respecting the structure of the urethra*," the least of any in the volume, because it is the least practical and most speculative of all. Its muscularity is here denied, but we do not find any thing proved more decisively than was proved before; the ground is still debateable, and therefore we shall confine our extracts to the following point of practice:

"So convinced am I of the impropriety of, and the bad effects which arise from, the use of bougies, in what are supposed to be cases of recently formed strictures, that whenever I am consulted by patients who have some difficulty in making water, and express their fear of the formation of a stricture, I invariably object to the introduction of the bougie, if, upon inquiry, I find that they have only recently recovered from gonorrhœa. My practice, in such cases, is to direct a small quantity of the Linim. Hydr. Camphor to be rubbed along the line of the urethra every night and morning; and it has always happened that those patients, who have had confidence to pursue this plan and give it a fair trial, have returned to me in the course of a short period, declaring themselves perfectly free from any obstruction or difficulty in making water.

"When obliged, for the satisfaction of my patient, to sound the urethra, in order to discover the situation of a supposed stricture, I have frequently observed, that after the bougie was passed three or four inches from the orifice, its point touching and pressing a particular spot, the patient has shrunk back, and expressed a feeling of pain. By a gentle pressure, however, the bougie may be passed onwards, and it afterwards proceeds with facility towards the neck of the bladder, without producing any unusual pain. On withdrawing the instrument, and pressing with the finger upon the urethra externally, at the part where the bougie produced the pain when in the passage, I have always found that the patient complained of soreness and a sensation different from what was caused by pressure upon any other part on the whole line of the canal.

"This is what some surgeons would term an incipient spasmodic stricture, requiring the daily use of the bougie to overcome its tendency to permanent contraction; but I have considered it as a remnant of the gonorrhœal inflammation; that this particular part of the membrane of the urethra continues inflamed, is consequently irritable, and requires a plan of treatment directly contrary to that which might be proper in a case of spasmodic stricture, supposing such an affection of the urethra to exist.

"In such cases, I direct the application of leeches as near as possible to the part affected, and a repetition of them at least once a week for a month, or sometimes longer, according to circumstances, prescribing at the same time full doses of the Liquor Potassæ, for the purpose of modifying the acrimony of the urine, and rendering it less stimulating to the inflamed surface over which it passes. When I find that the tenderness of the part has been removed by the local bleedings, and the patient no longer complains of soreness, or a dull indistinct pain on pressure, I advise the application of the Linim. Hydr. Camph. When this course of treatment has been diligently followed for some time, I have had the satisfaction of passing a full-sized bougie into the bladder, not only with the greatest facility, but also without causing the patient to suffer any unusual pain." (P. 38.)

The hernia cases are most of them very interesting, and very simply and sensibly detailed; we shall make extracts from one or two, and refer the reader to the volume for ample histories of the rest. Mr. Clement deprecates the use of tobacco enemata, which he thinks injurious to the patient, and states that *he has never known an operation succeed after the tobacco enema had been given.*

"*Case of Strangulated Umbilical Hernia, Operation followed by Enteritis; Death of the Patient.* Mrs. A., a very corpulent woman, upwards of seventy, had been subject to an umbilical hernia for many years, but she had never worn a truss, nor experienced any inconvenience from the rupture until the present time, July 13th, 1827. The tumor was generally flaccid, and by gentle pressure the greater part of its contents could be replaced within the abdomen.

"With the exception of a very torpid state of the bowels, which required the frequent use of purgative medicines, the patient, considering her age, enjoyed a good state of health, was of a remarkably cheerful disposition, and of very temperate habits.

"On the morning of July the 13th, when straining to evacuate the bowels, she felt that the hernia became larger and more tense than usual, and, on endeavouring to reduce its size, she was prevented by some uncommon resistance. She was shortly afterwards seized with acute pain around the umbilicus; this was succeeded by nausea and a disposition to vomit.

"She was bled freely, purgative medicines were prescribed, cold applications were directed to the tumor, and the taxis was long and carefully employed, but without success. The bowels were not relieved, no diminution in the size of the tumor was effected, and in the course of the evening the vomiting had become very distressing.

"July 14th. The symptoms continued during the night without any alteration. Two tobacco enemas had been administered, one in the night, the other early this morning. They produced great exhaustion which continued for some hours, but no good effect was derived from their use, as the hernia could not be reduced, the taxis again proving unsuccessful.

"At noon I was desired to visit the patient, and I found her suffering very acute pain: she complained of great soreness in the tumor, and tenderness all over the abdomen, which could not bear the slightest pressure. The vomiting was almost constant; and the ejected matter was very offensive, although not decidedly of a feculent nature. She had recovered from the effects of the tobacco enema, and the pulse had become quick and wiry. I abstracted sixteen ounces of blood, which was much buffed, and then attempted the taxis; but the tumor had become so tender that my efforts at reduction caused the greatest pain. I persevered for a short time, but became convinced that the operation, which afforded the only hope of relief, could no longer with propriety be delayed.

"I explained the nature of the case to the patient, a very intelligent lady, and, being aware of the danger of her situation, she most cheerfully acceded to my proposal, and even requested the immediate performance of the operation.

"In the presence of my father, Mr. Griffith, and some other professional gentlemen, I performed the operation at two o'clock in the afternoon. The tumor was very tense, and measured eleven inches in circumference; it was red and inflamed at the superior part, in consequence of the frequent attempts which had been made for its reduction.

"I considered, if an incision were made along the whole length of the rupture, that not only would there be an unnecessary exposure and handling of the intestine, but that the number of its convolutions might be found exceedingly troublesome, and embarrass me in the future steps of the operation. I therefore made an incision about three inches in length, first through the common integument, on the side of the tumor; and then gradually and cautiously raised layer and layer until the peritoneal sac was exposed: this was not dense and firm as I have generally seen it in other species of hernia, but thin and semi-transparent. An opening was carefully made into it, (when a quantity of serous fluid escaped,) and it was afterwards enlarged to the extent of the incision in the integument. I then passed my finger into the sac, and found that it contained only intestine, which was not adherent to any part of it.

"The stricture at the umbilical opening was firm and almost cartilaginous, and so narrow that it felt like a piece of packthread drawn closely round the intestine. This was more remarkable as the hernia was not of recent occurrence, and the patient had frequently been able to reduce the whole of its contents. The bistoury, with a sliding guard, was then introduced into the strictured opening, which I divided in a direction towards the linea semilunaris. The intestine was of a red colour, and so bright that it very much resembled a fine vermilion injection; I gradually returned one portion of it after another into the abdomen, until the sac was completely emptied.

"The wound was closed with sutures and adhesive plaster, a compress placed over it, and a roller applied moderately tight. The operation, although apparently long in the detail, was completed in a short time, and the patient bore it remarkably well.

"In the evening I visited her, and found the pulse very quick and wiry. She complains of excessive tenderness all over the abdomen. The roller was removed in the course of an hour after the operation, as its pressure became intolerable. Bowels have not been opened, and she has vomited once since the operation. I ordered her to be bled to sixteen ounces, a fomentation to be applied to the abdomen, and ten grains of calomel to be taken immediately. The nurse was directed to give the patient a dose of castor oil in the course of the night.

"July 15th, nine o'clock, A.M. She has past a very restless night, and complains of pain and soreness all over the abdomen. Bowels have not been opened. She vomited this morning, but not till some hours after the castor oil had been given. Pulse 110, and wiry; tongue much furred; respiration hurried. She cannot bear the slightest pressure on the abdomen. Fourteen ounces of blood were taken from the arm. Ordered thirty leeches to be applied, and an aperient draught in a state of effervescence to be taken every two or three hours.

"July 16th, eight o'clock, A.M. Although the bowels have acted once spontaneously since the enema was administered yesterday evening, there is no improvement in the condition of the patient. The vomiting and hiccough still continue, and the abomen is more tense. The pulse is fluttering, and so rapid as not to be easily counted.

"In the evening I again visited the patient, and found her, although perfectly sensible, sinking rapidly: the extremities were cold, and the hiccough very troublesome. She expired early the following morning.

"*Remarks.* Almost every practical writer in surgery has noticed how rarely the operation for strangulated umbilical hernia is attended with a successful result; and the present case affords an example of enteritis proceeding to a fatal termination, although attempts were made to arrest its progress by the most vigorous remedies.

"As the patient was a very corpulent woman, I expected to find some portion of the omentum in the sac, but it contained only a part of the small intestine, which was greatly distended with air. I have had frequent opportunities of dissecting umbilical hernias, and invariably found them to consist of portions of the colon, surrounded and enveloped, more or less, by the omentum; but I never observed in the dead subject any part of the small intestine contained in the sac.

"Two reasons induced me to make the first incision on the side of the tumor, and not along its upper or most prominent surface. The principal one was the inequality or irregularity of the peritoneal sac which is usually met with at the front of the rupture: indeed it generally happens that the several parts are so much consolidated, that the true sac cannot be easily distinguished, although I do not agree with some writers who suppose that it is entirely wanting, or does not extend upwards so far as to form an entire covering to the contents of the tumor. The second reason was, the red and inflamed condition of the integument covering the front of the rupture, produced by the repeated and long-continued use of the taxis. If an incision were made in that part, I thought the wound would not heal by the first intention, but in all probability would be followed by sloughing; a circumstance much to be dreaded, and more particularly in this species of hernia.

"I once saw a very celebrated surgeon operate in a case of large umbilical hernia: he made an incision along the whole length of the tumor, and afterwards opened the sac to its fullest extent. An immense quantity of omentum and intestine was immediately exposed; the convolutions of the latter were exceedingly troublesome; and I well remember that two pairs of hands were required to confine them before the operator could venture to divide the stricture, and when this was accomplished, a considerable length of time elapsed before every portion of the bowel could be replaced within the abdomen.

"The recollection of this case induced me to make an incision of such a length as would enable me to feel for, and divide, the stricture with facility, while at the same time there would be no unnecessary exposure of the contents of the hernia.

"In all cases of large umbilical hernia, requiring an operation, I should advise the surgeon to make his incision at the side of the tumor, and not along the front, as it will be nearer the strictured part, which can be more readily divided; and also because when any adhesion either of the omentum or intestine exists, I have found it to occur most frequently at the prominent or superior part of the rupture.

"This advice is contrary to the opinion expressed by Sir Astley Cooper, who recommends an incision to be made over the tumor in the form of a letter T inverted. It may appear presumptuous in me to differ from so great an authority; but, having repeatedly seen in dissection not only the sac and integument firmly united, but the intestine and omentum also inseparably adherent to the front or most prominent part of the tumor, I am convinced that, in a majority of cases, it would be impossible to perform the operation in the manner recommended by Sir A. Cooper without great danger of wounding the intestine.

"Although I am far from contending that adhesions do not occasionally exist at the sides of the rupture, still, as far as they are concerned, no greater difficulty will be met with in performing the operation, if the incision be made according to my recommendation, than the surgeon would have to contend with if he commenced his operation on the front of the tumor; while by this method he possesses the great advantage of being much nearer the seat of stricture, which can be divided without the necessity of exposing the whole contents of the hernia." (P. 99.)

The following is likewise interesting.

"*Case of Strangulated Femoral Hernia occurring in a Female near eighty years old; Total Want of Serum in the Sac; and remarkable Adhesion between the Omentum, the Sac, and the Intestine: the Operation Successful, after the Intestine had remained in a State of Strangulation seven days.* Mrs. Marston, aged seventy-seven, has had a reducible femoral hernia for three years, and, never having suffered any inconvenience from it previous to the present time, she has neglected the necessary precaution of wearing a truss. The patient is a remarkably thin and apparently weak woman, although, with the exception of her present illness, she has enjoyed a state of almost uninterrupted good health. She was capable of unusual bodily exertion for a woman at her time of life, and could undergo great fatigue, as was proved by the fact of her having walked nearly thirty miles in a day but a few weeks previous to the time when she was compelled to submit to the operation, the particulars of which I am about to relate.

"On Wednesday, the 29th of June, she was affected with very acute pain in the abdomen: her bowels had been constipated for some days previous; on Thursday vomiting commenced, and continued with little intermission up to Tuesday, the 5th of July, the time I first visited her.

"A variety of purgative medicines had been prescribed for her during the week, and several enemas were administered, without affording any relief, as the former were immediately rejected by the stomach, and the latter returned unaccompanied with any feculent matter.

"On my visiting the patient, I discovered that she had a very small femoral hernia, in a state of strangulation. The rupture was not very tense or painful when pressed upon; it did not rise up abruptly from under Poupart's ligament, so as to form a prominent and well-defined tumor, but it was compressed or flattened, and felt like an enlarged lymphatic gland advancing towards suppuration. At this time, the symptoms were very urgent; the patient was harassed by hiccough, and almost constant vomiting; her pulse was small, fluttering, and very rapid; the countenance anxious; and the surface of the body covered with a cold perspiration. The abdomen was not much distended, and pressure upon it did not cause any particular uneasiness, excepting when applied to the part immediately above the ligament of Poupart.

"Notwithstanding the tumor was soft and puffy, and the resistance not so great as is generally met with in cases where the contents of a hernia have remained very long in a strangulated condition, I found a reduction of the protruded parts quite impracticable; and I was obliged to relinquish the taxis after a careful and protracted trial.

"When I considered the advanced age of the patient, the great prostration of strength, and the length of time which had elapsed since the decisive symptoms of strangulation appeared, I did not feel justified in adopting any of those auxiliary methods which are generally employed to promote the success of the taxis. The alarming nature of the case having been explained to the patient and her friends, I strongly urged the necessity of an operation being immediately performed, as affording the only probable chance for her life. This proposal was cheerfully complied with, and the patient exhibited unusual firmness and resolution for one so far advanced in years and exhausted by her previous sufferings.

"The operation was performed at noon, in the presence of several of my professional friends. As the tumor was small, I made only one straight incision over its surface. The different layers of fasciæ covering the peritoneal sac were very distinct, and I experienced no difficulty in raising and dividing them. This part of the operation was a complete anatomical demonstration, and was rendered very simple and easy in consequence of the bloodless state of the wound. The true sac having been fairly exposed, the difficulty of the operation commenced. I found the sac adhering so firmly, and so intimately connected with the contents of the hernia, that to make an opening into it without endangering the parts below seemed almost impossible. I endeavoured to raise a portion of it upwards with my finger and thumb, so that I might use the scalpel horizontally, and with it make a small opening for the introduction of the director and bistoury. In this attempt I failed, and was equally unsuccessful with the forceps; the sac being inseparably attached to the whole anterior surface of the tumor, and resisting every effort made to raise any portion of it.

"Although the sac itself was considerably thickened, I felt distinctly that a portion of the omentum was lying uppermost, and the discovery of this gave me greater confidence in pursuing the operation. I scratched with the point of the scalpel, until the sac was divided, and a small portion of omentum protruded through the opening. I then attempted to introduce the director, but, in consequence of the close and firm adhesion existing between the sac and the omentum, I could not pass it upwards in such a manner as would leave only the sac between that instrument and the bistoury. I was therefore obliged to force the point of the director into the omentum; then to elevate it, and push it through its substance (taking care to direct the instrument as close as possible to the surface of the sac) until it arrived as near as I could guess at the seat of the stricture. The probe-pointed bistoury having been placed in the groove of the director, the sac and the most superficial portion of the omentum contained within it were freely divided. I then discovered that the omentum was also firmly attached to the sides and lower part of the sac: these adhesions I was obliged to tear asunder with my finger and the handle of the scalpel, before I could expose any part of the intestine. The gut was at length brought into view; it presented a very livid colour, but was so firm to the touch that I should have felt no hesitation as to the propriety of replacing it within the cavity of the abdomen, when liberated from the strangulation.

"The stricture was produced by the neck of the sac, which was divided without much difficulty, but in attempting to return the intestine I found that its lower surface was attached to the sac through the medium of coagulable lymph and two or three membranous bands. These adhesions prevented the free return of the intestine, and, for a moment, I hesitated whether to remain satisfied with the division of the stricture, and allow the intestine to remain in the sac, or to destroy these attachments, so as to be able to effect a reduction of the whole of the protruded parts. I resolved upon the latter plan, and having carefully divided the membranous bands, the intestine was very easily replaced within the abdomen.

"The patient was much exhausted by so protracted an operation; and, when placed in bed, I found it necessary to give her a powerful cordial. The extremities became cold, the pulse scarcely perceptible, and the prostration of strength so great as to threaten almost immediate dissolution. I directed bottles of warm water to be applied to the feet, and desired that she might take frequently some strong beef-tea, and a small quantity of brandy.

"I visited her at seven o'clock in the evening, and was happy to find that she had rallied considerably. The extremities had become warm, the pulse firmer, although still very rapid, the hiccough and vomiting had entirely ceased since the operation, and the patient no longer complained of any uneasiness in the bowels. I ordered the brandy to be discontinued, and in its place a little wine and water to be given every two or three hours.

"Wednesday, nine o'clock A.M. This morning there is a visible improvement in the condition of the patient. She slept nearly three hours during the night, and has remained quite free from any disposition to vomit. Pulse ninety-four; tongue much furred. The bowels have not been opened, but there is no tension nor tenderness of the abdomen. I desired the beef-tea to be continued, but the quantity of wine to be diminished.

"Seven o'clock P.M. She has taken freely of the beef-tea during the day, and is now cheerful, and expresses confidence in her recovery. Pulse eighty-six, and fuller than in the morning. The bowels have not been opened since the operation, I therefore ordered a dose of castor oil to be given immediately.

"Thursday, the 7th. I was desired to visit the patient early this morning. The castor oil had acted violently, producing eleven or twelve copious evacuations, and the patient is now apparently exhausted, and in as great danger as she was immediately after the performance of the operation. I was again obliged to have recourse to the brandy, and prescribed a mixture containing the volatile alkali and aromatic confection to be given every hour.

"Nine o'clock P.M. The bowels have not been acted on since nine o'clock this morning, and the patient has experienced the good effects of the stimulants which have been administered to her in the course of the day. The pulse continues small, but is not so rapid or fluttering as in the morning. I ordered a draught containing fifty drops of Tinct. Opii to be given at bedtime.

"Friday morning. The patient has passed a good night, and, but for the excessive debility she labours under, there is every prospect of a rapid recovery. The bowels have acted once this morning comfortably. There is no sickness, or tenderness of the abdomen. I removed the dressings, and found the wound almost closed, with the exception of the lower part.

"As no unusual symptoms occurred after Friday, it would be useless to transcribe from my notebook the further particulars relating to this case. The patient, notwithstanding her advanced age and great debility, was able to leave her bed in the course of a fortnight, and in less than a month after the performance of the operation she walked to my house, for the purpose of returning thanks." (P. 109.)

Cystocele is so rare a disease, that we shall make no apology for transferring the following to our pages. In No. 42, (N. S.) for December 1829 of our Journal, a somewhat similar case is recorded; but in which a post-mortem examination was not made.

"*Fatal Case of Cystocele, or Hernia of the Bladder, in which the whole of that Viscus had escaped from the Pelvis, and was lodged in the Scrotum.* The subject of this uncommon affection was a Mr. Bowley, formerly residing in Shrewsbury, a very corpulent man, and upwards of sixty years of age. He had been affected with a scrotal hernia for five and twenty years, which, during that long period, had never been productive of any other inconvenience (until a short time previous to his death,) than what might have been expected from its great bulk and weight.

"The patient had repeatedly suffered from constipation of the bowels, and slight attacks of hemiplegia, which, however, yielded to the ordinary remedies prescribed in such cases; his general health in other respects was good, and he was accustomed to take regular, occasionally laborious, exercise.

"It was only a fortnight prior to the death of the patient that the hernia appeared to produce any alarming or dangerous effects, when the bowels became obstinately constipated, and he was attacked with paralysis which affected the left side of the body. Conjoined with these symptoms was a constant stillicidium urinæ; the catheter was used several times, and according to the belief of two surgeons who were in attendance, passed into the bladder, but not more than a teacupful of urine followed each introduction of the instrument. The cause of this, although unfortunately not ascertained during the lifetime of the patient, was afterwards most satisfactorily explained by the dissection.

"The patient was never affected with any of the urgent symptoms commonly attendant on strangulation of the intestine, at least they were not so decided and unequivocal as to justify the performance of an operation in the opinion of the gentlemen who attended the case.

"The first object in the treatment was to procure relief for the bowels, and every method was adopted for that purpose without success. The strongest purgatives were given, and enemas repeatedly administered, without producing any beneficial effects.

"As the patient was deprived of the power of voluntary motion, by the paralysis which affected the whole of the left side of the body, his bladder was supposed also to be paralysed; and the medical attendants considered that this opinion was confirmed by the continuance of the stillicidium urinæ.

"Although no evacuation could be produced by the use of purgatives, or by the administration of the enemas, the patient for several days laboured under symptoms which seemed to be more dependent upon retention of urine than constipation of the bowels: he complained of great pain about the pubes, and in the rupture, which became gradually more distended. At length the power of articulation (before much impaired) was totally lost; the sufferer could express his feelings and wants only in a low muttering tone; he shortly afterwards became delirious, and died.

"The foregoing is a slight but correct history of the general symptoms of this case, with the treatment of which I was not concerned. I was present at the examination of the body, which was made twenty-four hours after death.

"*Appearances on Dissection.* The circumference of the rupture was two feet five inches; its greatest length, measuring in a direction over the pubes to the apex of the tumor, one foot two inches and three quarters. The whole of the penis was retracted within the integument covering the rupture; the opening through which the urine flowed very much resembled the navel, and this gave to the tumor the appearance of an immense umbilical hernia, extending over the pubes and falling down between the thighs. One of the testicles could be distinctly felt near the surface, about the middle of the tumor, but the other was not discoverable before the parts were dissected.

" Although the rupture was so large as to extend generally over the pubes, and occupy both inguinal regions; it was easily ascertained, without making any incision, that the protruded parts came down through the left abdominal ring.

" The examination was commenced by dissecting for the left inguinal, which was exposed, and part of the colon found passing through it greatly distended with feculent matter, but exhibiting no marks of inflammation or strangulation: the latter could not have taken place, the passage being so much dilated as to give the whole hand free admission into the cavity of the abdomen.

" A semicircular cut was next made through the integument, following the course which the colon had taken downwards in the sac; this incision was continued some inches in length, when one of the testicles was exposed, and we were surprised by the sight of another distinct sac, very tense and containing fluid. By this we were led to suppose that some portion of the intestine must also have protruded through the abdominal ring on the right side, forming a double kind of hernia, This part was again carefully examined, but no intestine could be traced to that side: besides, the surface of this second or supposed sac was too regular to contain either omentum or intestine; it formed the greatest bulk of the whole tumor, and more resembled a hydrocele, if it was possible to conceive one to attain a size so immense. By dissecting the integument from the surface, the apex of this second sac was found to be very thin, red, and pointed, in fact, appearing ready to burst: this inflamed part was accidentally ruptured, and about two quarts of very fetid urine was evacuated, which removed all doubt and previous uncertainty as to its nature.

" By following down the course of the urethra, which was wonderfully displaced from its natural situation, we discovered that the bladder of urine had protruded through the abdominal ring. Owing to the prejudices of the patient's friends, all the parts could not be brought away, so as to make a complete preparation shewing thet relative situation which they observed to each other. The bladder was however removed from the body, and, upon examination of the prostate gland, which was much enlarged, I found that it had been perforated by the catheter, in the ineffectual attempts made to draw off the urine. The diameter of the ureters was so much enlarged as to admit my forefinger with great facility.

" The abdomen presented no marks of acute inflammation having recently taken place; the omentum was much loaded with fat, and the whole length of the colon was greatly distended with feculent matter.

" In the pelvis there was nothing remarkable excepting the want of the urinary bladder; the natural connexion which exists between that viscus and the inner surface of the pubes was not to be discovered." (P. 145.)

"*A Tumor of an uncommon Nature, situated in the Biceps Flexor Cubiti.* In an early number of the Medical and Physical Journal, Mr. Symmonds, of Manchester, has related some cases of bony concretions formed in, and connected with, different parts of the body, which, from the obvious dissimilarity of structure, there could have been no previous or natural relationship.

"The following case resembles in some particulars those published by Mr. Symmonds.

"John Wheeler, a young man, by trade a shoemaker, consulted me respecting a tumor upon his arm, which latterly had produced much uneasiness, and occasionally severe pain. It was not very prominent; was situated over the belly of the biceps flexor muscle; was excessively hard, and conveyed to the fingers much the same feeling as the upper portion of a fractured patella when retracted by the action of the extensor muscles. Although apparently superficial, its lower surface was firmly attached to the muscle, and whenever the forearm was bent suddenly upon the humerus, the patient complained of acute pain.

"On inquiring into the history of the case, the patient informed me that for many years be had a small 'knot' on his arm, which, however, was not attended with any inconvenience. About two years previous to my seeing him, this substance began to increase, and during the last nine months its growth had been very rapid, although several applications had been made with the view of retarding it.

"Having explained to the patient that the tumor was of that peculiarly hard structure not likely to yield to any external application, I advised him to submit to its extirpation, as it totally incapacitated him from following his trade: but he dreaded the knife, and would not submit to any operation. I accordingly dismissed him, as I could not promise him relief by any other method. In the course of a few weeks, however, the patient returned, and desired me to operate.

"As the integument was loose, and not adherent to the upper surface of the tumor, I made a rather long incision over it, and dissected down to its substance, which felt gritty under the knife. The edges of the wound being then drawn asunder, I seized the tumor with my fingers, and endeavoured to elevate it from its situation; but I found it so firmly attached to the muscle, that I was obliged to dissect deep into the fibres before its complete separation was effected. When removed, the lower part of the tumor was found to be of a conical shape, and very irregular, whereas the surface in contact with the integument was flat and rather smooth.

"The sides of the incision were drawn together by adhesive plaster, and, when the dressings were removed, on the fifth day after the operation, the wound had healed by the first intention.

"The examination of the tumor proved it to consist of a dense cartilaginous mass, intersected by, and for the most part composed of, particles, which I know not how to describe most accurately, whether by calling them of an osseous or calculous nature. The colour of these gritty particles was varied; in some parts they were as white as chalk, but in the centre of the tumor of a yellow tinge. The tumor was not surrounded by any proper investing capsule; and in no part of it could I detect any appearance of vascularity; but portions of the muscular fibres were so firmly attached to the irregularities of its lower surface that it was with great difficulty they were separated: the portions of flesh were imbedded in the crevices of the tumor and connected with it, much in the same manner as the pulp of the fruit is attached to the unequal surface of a peach stone." (P. 193.)

We have made more copious extracts than we at first intended, but we are far from having exhausted the volume, which our readers still may peruse with satisfaction rather increased than diminished by the samples we have afforded. The getting up of the book is on the whole very creditable to the provincial press: some errors, however, remain, from carelessness in correcting the proof-sheets. In one French quotation, for example, at page 18, there are almost as many blunders as there are lines.

---

*The Microscopic Cabinet of select Animated Objects; with a Description of the Jewel and Doublet Microscope, Test Objects, &c. To which are subjoined, Memoirs on the Verification of Microscopic Phenomena, and an exact Method of Appreciating the Quality of Microscopes and Engiscopes;* by C. R. Goring, M.D. *Illustrated, from original Drawings, by thirteen coloured Plates and numerous Engravings on Wood.* By Andrew Pritchard.—8vo. pp. 246; thirteen plates. Whittaker and Co. London.

Dr. Goring and Mr. Pritchard are already favorably known to the philosophic world for their unwearied diligence, and the success which has crowned their exertions, devoted as both have been to the improvement of microscopes; the one as a very ingenious artist, the other not only as a fellow-labourer in the field of science, but also as a most liberal patron. Mr. Pritchard, we need hardly advertise our reader, is the maker of jewel microscopes, and the first person who ground diamonds into lenses, and the first who ever used gems as magnifiers, to which he was led, as he very properly acknowledges, by Dr. Goring, by whom the first diamond was supplied to be converted into a lens, and by whom the artists consulted and employed were furnished with the means of procuring such new tools and appurtenances as were required for this new process; but we could have been well pleased had Mr. P. likewise acknowledged the part which Mr. Varley,* whose apprentice he then was, had both in the mental and manual operations, whose liberality was on that, as on many other occasions, worthy approbation: we assure him he would not, by so doing, have lessened that credit which is undoubtedly his own.

The importance of diamond and jewel lenses for miscrocopes is now confessed on every side; we therefore need say nothing in their commendation; it might, however, have been supposed that the use of such substances as diamonds for magnifiers would, from their costliness, have rendered them matters rather of curiosity than of practical utility; but, on the contrary, their durability is so much greater than lenses made of glass, that their extra cost is scarcely worth computing; and, in the end, it is probable they will be found the most economical of any.

For the detailed description of the microscopes and engiscopes here recommended, and also for Dr. Goring's very valuable memoirs "concerning the Verification of Microscopic Phenomena, with Fruits of Experience relative to the Analysis of Test Objects, and the Defining and Penetrating Powers of Microscopes and Engiscopes," and "on the Exact Method of Appreciating their Quality," we must refer all that are interested in these matters (and who is not?) to the volume, where we can promise that they will find much very important information. We cannot, however, refrain from extracting Pritchard's account of his first essays in grinding diamonds into magnifiers.

"The valuable properties of the diamond for microscopic purposes, were first pointed out by Dr. Brewster, in his admirable Treatise on new Philosophical Instruments, published in 1811, in which the following passages occur, though it seems the Doctor never contemplated the possibility of working this refractory substance into magnifiers.

"'In all minerals, in which a metal is the principal ingredient, those which have the greatest density have also the greatest faculty of producing colour, while in all the precious stones a high refracting power is attended with a low dispersive power," p. 314; "we cannot therefore expect any essential improvement in the single microscope, unless from the discovery of some transparent substance, which, like the diamond, combines a high refractive with a low dispersive power,' p. 403.

"In the summer of 1824, Dr. Goring directed my attention to the above passages, of which I immediately saw the full force, and it was agreed that I should undertake to grind a diamond into a magnifier. For this purpose, Dr. Goring forwarded me a small brilliant diamond to begin upon, and it was proposed to give it the

* Mr. Varley has lately constructed a microscope that seems peculiarly well adapted for the examination of animalculæ, and which we did intend to have noticed in the present number, but space compels us to reserve our observations till the next.

curves that in glass would produce a lens of a twentieth of an inch focus, with the proportion of the radii of their surfaces, as two to five. This stone I ground with the proper curves, and polished the flatter side, contrary to the expectations of many, whose judgment in these matters was thought of much weight, who predicted that the crystalline structure of the diamond would not permit it to receive a spherical figure. When thus far advanced, fate decreed that I should lose the stone, and my only consolation was to discover afterwards that, had it been completed, its thickness and enormous refractive power would probably have caused the focus to fall within the substance of the stone.

" Having, however, in this experiment proved the possibility of working lenses of adamant, I set about another, and selected a rose-cut diamond, in order to form it into a plano-convex lens, and thereby save a moiety of the labour.

" In the progress of working this stone, the heat generated by friction in the course of the abrasion of the diamond was perpetually melting the cement (shell-lac), by which the flat side was affixed to the tool, and compelled me to seek some means by which it might be prevented. After several trials, I found that when a portion of finely-powdered pumice-stone was mixed with the shell-lac, the cement was much stronger, and less liable to melt than any other similar substance.

" On the 1st of December, 1824, I had the pleasure of first looking through a diamond microscope, and it was doubtless the first time this precious gem had been employed in making manifest the hidden secrets of nature. A few days after, I had polished it sufficiently to put it into the hands of Dr. Goring, who tried its performance on various objects, both as a single microscope, and as the objective of a compound. He states, in a letter addressed to me, dated 3d of January, 1825, that 'it has shewn the most difficult transparent objects I have submitted to it;' and again, 'I can clearly perceive the amazing superiority it will possess when completely finished.' I must, however, inform my readers, that we discovered in this state various flaws in the stone, in consequence of which we abandoned all thought of completing it.

" In this condition the project remained for about a year, when I determined to resume my attempts, and, having worked several stones into lenses, I at last succeeded in obtaining a perfect one. In the course of these labours, a new, though not unexpected defect, appeared in several lenses, which would have subverted the whole scheme, had not the first diamond lens been free from it.

" These lenses, instead of giving a single image like the first, gave a double or triple one. This rendered them utterly useless as magnifiers, and made the defects of soft and hard parts in the same stone, and the small cavities in others, of comparatively trifling consequence. The images exhibited in such lenses overlapped each other, but were never entirely separated, though the quantity of overlapping varied in different specimens.

"It was now evident that these defects arose from polarization, though this stone is described as 'refracting single.' I subsequently learnt, from Dr. Brewster, after I had overcome these obstacles, that this property of the diamond had been observed by him, and an account of it given in the Edinburgh Philosophical Transactions. On referring to his paper, it appears Dr. B. found that some stones 'polarized in particular parts, while other portions of the same stone were quite free from any trace of polarity,' and thus perfectly adapted to our purpose, as had previously been demonstrated in the first diamond lens.

"Notwithstanding these difficulties, and the consequent expense and labour they entailed on me before sufficiently experienced in working upon this refractory material with certainty, I have now the satisfaction of being able, by inspection *a priori*, to decide whether a diamond is fit for a magnifier or not, and have now executed two plano-convex magnifiers of adamant, whose structure is quite perfect for microscopic purposes. One of these is about the twentieth of an inch focus, and is now in the possession of his Grace the Duke of Buckingham; the other, in my hands, is the thirtieth of an inch focus, and has consequently amplification enough for most practical purposes." (P. 107.)

"It must be perceived, from what is stated above, that the chief impediment to the introduction of diamond microscopes is their vast expense; but, as their durability exceeds that of any other substance from which magnifiers can be formed, it is questionable whether in the end they are more costly than glass. Such being the case, my attention was next directed to obtain a substitute that might be procured at a cheaper original cost than the diamond. Taking this view of the subject, it occurred to me that the sapphire, or ruby, might be employed with advantage, and at an outlay not much exceeding high powers of glass, which the result has verified." (P. 111.)

Many of the descriptions of the structure and habits of animals, as observed and described by our author, will be found curiously interesting; we select two or three passages from the earlier chapters, as specimens: and first of the "*Larva of a small Species of Dytiscus, popularly called the Crocodile.*"

"This larva is armed with a pair of bent forceps or mandibles, as shewn in the engraving; they move horizontally, and are long enough to cross each other when closed; they are of a bright chesnut colour, assuming a deeper hue towards the points, which are hard and sharp. With these weapons it seizes its prey, and, having brought it towards the mouth, it commences the operation of exhausting the juices from that portion within its grasp, having previously made an incision with the mandibles. This larva does not kill its victim before eating it, unless compelled by the superior strength of its prey, but, taking hold of any part indiscriminately, it devours that portion while the animal is alive: having so done, if its victim be the larva of a gnat, or other soft animal, it turns it round, and thus brings a fresh portion within its grasp, alternately opening and closing each mandible till the whole is consumed, except the skin. If the prey is a strong crustaceous animal, it seizes it, and either holds it for some time stationary, till it is exhausted, or nips off, at successive grasps, all its limbs, turns it upon its back, and imbibes its contents." (P. 22.)

"These larvæ feed on almost every other kind, and in their turn, are devoured by the larger water-beetles, (Dyticus Marginalis and Semistriatus.) Their favorite food is the larvæ of the emphemera and gnat. On the other hand, even when kept without food, they refuse monoculi, preferring to feed on each other. From this propensity, if confined in separate vessels for a few days, and afterwards put together, the most fierce and obstinate combats ensue. In these engagements the little animals display all the courage, skill, and caution of two well-trained pugilists, turning about with extended jaws, till a fit opportunity occurs for attack. Their courage is such, that I have seen a small one seize another twice its size, and hold it for several seconds. When, however, they are of equal size, and exceedingly pressed by hunger, the contest will be continued for several minutes, and, to the lovers of such sports, will be found not inferior to any, and, when viewed on the screen of a solar microscope, several spectators can witness it at the same time, and make their observations and remarks as the battle proceeds." (P. 24.)

The history of the larva and pupa of a species of dragon-fly, (Æshna grandis,) is likewise well worthy perusal; we regret that we cannot do more than select a paragraph or two from this chapter.

"The eyes of these creatures are very prominent, both in the larva and final state; and from their size and curious structure, afford excellent objects for miscroscopic examination. In the perfect insect they have been a fruitful object of study to naturalists. They are immoveably fixed on each side of the head, and are compound, each consisting of numerous distinct smaller ones. They are externally convex, and it has been observed by Latreille, that the eyes of insects in general, are 'by so much the more convex as the insect is more carnassial.' Under a low magnifier the surface appears reticulated, which, on minute examination, is found to arise from hexagonal cells; each forming a separate eye. Leeuwenhoek states, that he has counted twelve thousand in one individual. The cornea consists of lenses possessing all the properties of those made of the usual transparent media, forming an image of bodies in the same manner, and capable of being employed as magnifiers. These interesting facts may be observed, by placing any object under the eye of the insect, and viewing it in a miscroscope, when each of the minute lenses of the eye will form an inverted image of the object employed. By separating one of these lenses, and forming an inverted telescope with it, using a magnifier of low power as an eye-glass, and the eye of the insect as the object-glass, and adjusting their distance, a distinct view of objects at a moderate distance may be readily obtained. In this way, the focus of the eye may be found, as in the case of common lenses, if we know the exact power of the eye-glass: for example, if this magnifier is the one twentieth of an inch, and on looking through this inverted telescope at the window bars, you find (keeping both eyes open) that three of the squares of glass are exactly equal in length and breadth to one seen by the other eye at the same time without the telescope, the two images being brought apparently to overlap each other, the focal length of the eye under examination will be one third of the eye-glass, or one sixtieth of an inch. I regret that I have not measured the focal length of the eye we have been describing, but in the common house fly (musca domestica), the lenses are each about the one hundredth of an inch focus. In preparing the compound eyes of insects, it is requisite to soak them for some days in water, to render them supple, and then to wash out the black pulp (rete mucosum), with a camel's hair pencil, when they may be mounted between slips of glass, or in ivory sliders, unless it be intended to measure the exact focus, when all pressure must be avoided.*" (P. 41.)

"The transformation of the pupa to the perfect fly, is accomplished in the short space of a few minutes, and is an occurrence that is seldom observed. At the period of the change, it crawls out of the water, and fixes itself by its claws to some adjacent plant, and after remaining a few seconds it becomes dry, and the skin along the back separates, allowing the head and legs and part of the body of the perfect insect to be protruded, while the empty skin of the feet remains firmly fixed to the plant; it now remains stationary for a short time longer, while the wings expand and unfold themselves; the remaining parts are then liberated, and when sufficiently extended and dry, the perfect fly soars into the atmosphere." (P. 44.)

Mr. Pritchard thinks, from certain phenomena which he has observed, that the common polypes *Hydra viridis* and *grisea*, like the torpedo and gymnotus, have the power of communicating an electrical shock: this, if true, would be very curious, seeing that their nervous system (?) is altogether in embryo, and their structure so very simple. He says of these animals:

"When in search of prey, they stretch out their arms and body to the utmost, and spread the former in various directions, thus presenting a large surface to entrap it. As soon as an animal comes within their range, they entwine their arms about it, and afterwards, by contracting them, bring it to the mouth and devour it. It

* "It appears, from some recent dissections of compound eyes, that the plates which compose the cornea are distinct lenses, each capable of forming an image; a tube is placed behind them, and another lens at the opposite end. A favorable example of this construction is the pedunculated eye of the crawfish and lobster. It is difficult to find a subject more interesting for microscopic investigation than the dissections of compound eyes."

sometimes happens that the velocity with which their prey is moving prevents the polype from securing its victim. In such cases I have observed the little animal, after the attack, sink in the water apparently lifeless, and remain so for a few seconds before it resumed its wonted activity. From this singular fact, I am induced to imagine that the polype possess the power of giving minute electric shocks, similar to some fish and insects; for in no other way can I account for the momentary torpor of such active little animals, as the water-fleas (Daphnia Pulex) and the Cyclops, after coming within its reach; and this is the more probable, as their prey, even when it consists of worms (whose tenacity for life is well known), is instantly deprived of life, and no weapons of any kind can be discovered." (P. 72.)

As a verification of a disputed point we insert the following:

"The most remarkable fact relative to these creatures, is, that they can be cut asunder, and each part will reproduce the deficient parts, and form a perfect animal. The best account I have seen of experiments on this subject is given by Baker, in his 'Natural History of the Polype,' before alluded to. His results, however, have been discredited by several intelligent observers; I have therefore made some experiments on them myself, and find them agree with his statements in every material point: a description of the details of one may not be uninteresting.

"On the 22d of August I selected a brown polype out of a glass vase containing a good supply of them, none of which had more than seven arms. The individual selected was then laid on a plate of glass, with a drop of clean water. Having provided myself with a good Whitechapel needle, and ground the pointed end so as to form a cutting edge, I severed the polype obliquely, the superior part comprising the greater portion of the head and four arms; the inferior part being the tail, and the remainder of the head with two arms. These pieces were then put into a four-ounce phial of water, with a few small crustacea (Daphnia and Cyclops), where they sunk to the bottom apparently lifeless. Three hours after the operation I examined them (without disturbing the vessel), and found them in the same inert state. Twelve hours after this, I found the inferior piece attached to the side of the phial by its tail, with his arms extended in quest of food, the superior one still remaining at the bottom, but with its arms extended like the other. On the 24th, a new tail was completed to the superior portion of the polype, and the rudiments of additional arms were developed in both; they appeared in good health. The third day the new arms were nearly of the same size as the others, and in less than a week each polype had a young one shooting from it.

"The most curious circumstance connected with this experiment, was that the two new polype had each ten arms, while that from which they were produced, as well as those that were in the same vessel, had only six or seven." (P. 74.)

With the following account of the Lurio, or glutton, we must, however unwillingly, conclude our extracts.

"The œsophagus connecting the cavity of the mouth with the first stomach, is capable of great and instantaneous expansion, and is never completely closed, for its prey, which it always swallows alive, may be observed moving about in the first cavity, and endeavouring to make its escape through the contracted opening. All the digestive cavities or stomachs are preserved in their proper situation by a transparent muscular annulus between each of them. These diaphragms possess considerable contractile power, and are attached by their outer circumference to the muscular stratum, under the skin of the animal, while their inner margin surrounds the contracted parts of the alimentary canal, and is fastened to it. The fibres of these muscular plates diverge like rays, analogous to those in the iris of the human eye, and vary the aperture of the stomach, like the pupil of the eye, in the latter case." (P. 80.)

"The digestive power of the stomachs must be very considerable, as the food which they prefer is crustaceous. They will often devour monoculi greater in diameter than their own bodies, and that with a degree of rapidity and insatiability not inferior to the Boa constrictor, with whose manner they assimilate. It moves its head with a sluggish motion, and, when filled to repletion, is altogether inactive. The mouth is not possessed of any organs for mastication, nor has it any weapons of defence. So great is the voracity of this creature, that I have seen a middle-sized one devour seven Lyncei in half an hour. Five of these were moving about in the first cavity, at the end of that time; the other two, having passed into the second, had become exhausted." (P. 81.)

The style in which this work is got up is very creditable, and the illustrations extremely good and copious; for, besides the seventeen plates, there are numerous woodcuts introduced among the text; and we trust that the labour bestowed upon the book, both in matter and materials, will not go unrewarded.

